# Textbook oncologic outcomes are associated with increased overall survival in patients with pancreatic head cancer after undergoing laparoscopic pancreaticoduodenectomy

**DOI:** 10.1186/s12957-024-03322-8

**Published:** 2024-02-06

**Authors:** Jing Zhang, He Cai, Man Zhang, Xin Wang, Yunqiang Cai, Bing Peng

**Affiliations:** 1 Department of General Surgery, Division of Pancreatic Surgery, West China Hospital, Sichuan University, No. 37, Guo Xue Xiang, Chengdu, 610041 Sichuan China; 2 Department of Minimal Invasive Surgery, Shangjin Nanfu Hospital, Chengdu, China; 3 The Health Management Center of West China Hospital, Sichuan University, Chengdu, China

**Keywords:** Laparoscopic pancreaticoduodenectomy, Pancreatic head cancer, Textbook oncologic outcome, Prognosis

## Abstract

**Background:**

Textbook oncologic outcomes (TOO) have been used to evaluate long-term oncologic outcomes for patients after pancreaticoduodenectomy (PD) but not laparoscopic pancreaticoduodenectomy (LPD). The aim of the study was to assess the prognostic value of TOO for patients with pancreatic head cancer undergoing LPD and discuss the risk factors associated with achieving TOO.

**Methods:**

Patients with pancreatic head cancer who underwent LPD in West China Hospital from January 2015 to May 2022 were consecutively enrolled. TOO was defined as achieving R0 resection, examination of ≥ 12 lymph nodes, no prolonged length of stay, no 30-day readmission/death, and receiving adjuvant chemotherapy. Survival analysis was used to determine the prognostic value of a TOO on overall survival (OS) and recurrence-free survival (RFS). Logistic regression was used to identify the risk factors of a TOO. The rates of a TOO and of each indicator were compared in patients who suffered or not from delayed gastric emptying (DGE).

**Results:**

A total of 44 (25.73%) patients achieved TOO which was associated with improved median OS (TOO 32 months vs. non-TOO 20 months, *P* = 0.034) and a better RFS (TOO 19 months vs. non-TOO 13 months,* P* = 0.053). Patients suffering from DGE [odds ratio (OR) 4.045, 95% CI 1.151–14.214, *P* = 0.029] were independent risk factors for TOO. In addition, patients with DGE after surgery had a significantly lower rate of TOO (*P* = 0.015) than patients without DGE.

**Conclusions:**

As there were significant differences between patients who achieved TOO or not, TOO is a good indicator for long-term oncologic outcomes in patients with pancreatic head cancer after undergoing LPD. DGE is the risk factor for achieving TOO, so it is important to prevent the DGE after LPD to improve the rate of TOO.

## Introduction

Pancreatic ductal adenocarcinoma (PDAC) is one of the most malignant diseases, and surgical resection is the only possible cure treatment [1]. For patients with PDAC in the location of the pancreatic head, pancreaticoduodenectomy (PD) was a standard procedure [2]. With the development of minimal invasive surgery, laparoscopic pancreaticoduodenectomy (LPD) was first proposed by Gagner and Pomp in 1994 [3] and has been shown to have the advantages of less blood loss, faster recovery, and less pain compared with open PD [4]. As for the long-term outcomes, there is some controversy, especially in treating PDAC. Overall, in many previous published studies, there is no significant difference in the overall survival (OS) for PDAC compared with open PD [2]. For managing these patients, complex multidisciplinary care and using indicators to predict long-term survival are very important and useful [5].

However, individual indicators such as morbidity, length of stay (LOS), and readmission often cannot fully reflect the surgical quality metrics and predict the long-term prognosis [6]. Since its introduction by the Dutch Colorectal Consortium in 2012, the concept of “textbook outcome” (TO) as a comprehensive quality metric has been embraced by many patient-centered researchers seeking to define the “best” disease or specific surgery [7, 8]. Like TO, the textbook oncologic outcome (TOO) has been used in colon, stomach, liver, and esophageal cancer, for the comprehensive assessment of the surgical treatment by previous researchers [9–14]. The definition of TOO may be useful in evaluating the treatment of complex disease processes, such as pancreas cancer [5].

Previous studies have demonstrated that TOO is associated with survival in a variety of tumors, including pancreatic cancer, liver cancer, and esophageal cancer [5, 13, 14]. However, studies on patients with pancreatic cancer mainly focus on open surgery, but there is no relevant exploration into laparoscopic surgery. Thus, in this study, we would like to explore the association between TOO and long-term oncologic outcomes like OS and recurrence-free survival (RFS) in patients with pancreatic head cancer after undergoing LPD. In addition, we identified the risk factor for TOO.

## Methods

### Study design and patients

We conducted this retrospective case–control study at a high-volume laparoscopic surgery center, the West China Hospital, in which all the operators had passed the learning curve. The eligibility criteria of the patients for this study were as follows: (1) the consecutive patients underwent LPD for pancreatic head cancer from January 2015 to May 2022, (2) the postoperative pathological diagnosis of the patient was PDAC, (3) the perioperative and follow-up data information can be collected completely, and (4) patients without the history of neoadjuvant chemotherapy or radiotherapy. The exclusion criteria were as follows: (1) cases conversed to laparotomy due to various reasons during operation, (2) patients with borderline resectable or locally advanced status determined by abdominal vascular enhanced computed tomography (CT) preoperatively according to the NCCN guidelines [15], and (3) patients underwent open surgery.

We grouped the patients who achieved all six criteria of a TOO to the TOO group and either to the non-TOO group. TOO was defined when all six individual oncology criteria were met: negative resection margins, standard number of lymph nodes dissected as defined by the American Joint Committee on Cancer (AJCC), no prolonged LOS (LOS ≤ 50 percentage points), no 30-day unexpected readmission, no 30-day postoperative death, and adjuvant systemic chemotherapy was started within 12 weeks of surgery. The standard number of lymph node dissection in pancreatic cancer patients defined by AJCC was ≥ 12 lymph nodes [16]. Postoperative adjuvant systemic chemotherapy was based on the recommendations of the NCCN guidelines [15]. The personal information of patients in this study was replaced by coding to conceal private information, which has passed the ethical review of West China Hospital of Sichuan University.

### Data collection

Patients’ demographic data were collected including age, sex, body mass index, and laboratory test. Operative information included operative time (OT), estimated blood loss (EBL), and intraoperative blood transfusion. Postoperative outcomes such as short-term complications, 30-day mortality, incidence of readmissions, and LOS were recorded. To be defined as a postoperative pancreatic fistula (POPF), we strictly followed the International Study Group of Pancreatic Surgery (ISGPS) suggestion that drain fluid on or after postoperative day 3 with amylase level > 3 times the upper limit of normal amylase for each specific institution is the necessary threshold, and this condition needs to be clinically relevant [17]. Delayed gastric emptying (DGE) represents the inability to return to a standard diet by the end of the first postoperative week and includes prolonged nasogastric intubation of the patients [18], and post-pancreatectomy hemorrhage (PPH) is defined by ISGPS according to the site of bleeding, severity, and clinical impact [19]. Follow-up was performed at 3-month intervals for the first 2 years after surgery. If the patient shows no signs of recurrence 2 years after surgery, the follow-up examination is changed to a 4–6-month interval. Recurrence was determined based on radiological evidence (abdominal CT or PET/CT) or level of CA19-9 and other tumor markers in the serum. Follow-up was mainly conducted by telephone and through outpatient rechecks; other information was obtained by medical records and population death register information system. All data of this study was collected and checked retrospectively by two investigators from the prospective maintained medical record system, and any inconsistency needs to be discussed to get agreement.

### Surgical procedure and perioperative management

Patients were diagnosed with pancreatic cancer by abdominal enhanced CT, tumor markers in the serum, and sometimes by ultrasound-guided puncture. According to the NCCN guidelines, non-metastatic pancreatic cancer is classified as resectable, borderline resectable, or locally advanced based on the extent of vascular involvement [15]. For patients with resectable pancreatic cancer, we prefer to perform LPD. For patients with a tumor diameter larger than 5 cm and with two or more previous upper abdominal operations, we would decide whether to convert to open surgery according to the results of laparoscopic exploration, and we would recommend patients with borderline resectable pancreatic cancer to undergo neoadjuvant therapy first. Surgical procedures and perioperative management of LPD were described in detail in our previous reports of our center [20–23]. When necessary, portal vein or superior mesenteric vein (PV/SMV) resection and reconstruction were performed [24]. A nasogastric tube was used during surgery, removed 1–2 days after surgery, and taken orally if tolerated. Serum and drainage amylase were routinely assessed on postoperative days 1, 3, 5, and 7. Then, thoracic and abdominal CT were reexamined 4–5 days after surgery. When CT showed no abnormal findings, abdominal drainage was removed in patients [25]. The postoperative adjuvant systemic chemotherapy was formulated according to the NCCN guidelines [15] and started as soon as possible according to the patient’s recovery and willingness.

### Statistical analysis

Baseline data, tumor characteristics, and postoperative complications were presented as continuous and categorical variables. The missing data was processed by sequential regression multiple imputation. Then one-sample Kolmogorov–Smirnov test was used to assess whether the continuous variables were following normal distribution. For continuous variables following normal distribution, the mean ± standard deviation (SD) was reported and tested by the Student *t* test. Otherwise, the median with interquartile range (IQR) was reported and tested by independent samples Mann–Whitney *U* test. Descriptive statistics for categorical variables were reported as frequency and percentage and assessed using the Pearson chi-square test or Fisher’s exact test depending on the expected count. Survival curves of OS and RFS were plotted using the Kaplan–Meier method to determine the effect of TOO on survival. The equality of functions was assessed using the log-rank test. Multivariate logistics regression analysis was performed to determine which variables were independently associated with achieving TOO outcomes, and variables with *P* < 0.10 in univariate analysis were included in multivariate analysis. To explore the implementation of each criterion in TOO, we draw a summary histogram. All analyses were performed using IBM SPSS Statistics 26 (IBM Corp., Armonk, NY).

## Results

### TOO and cohort characteristics

**Fig. 1:**
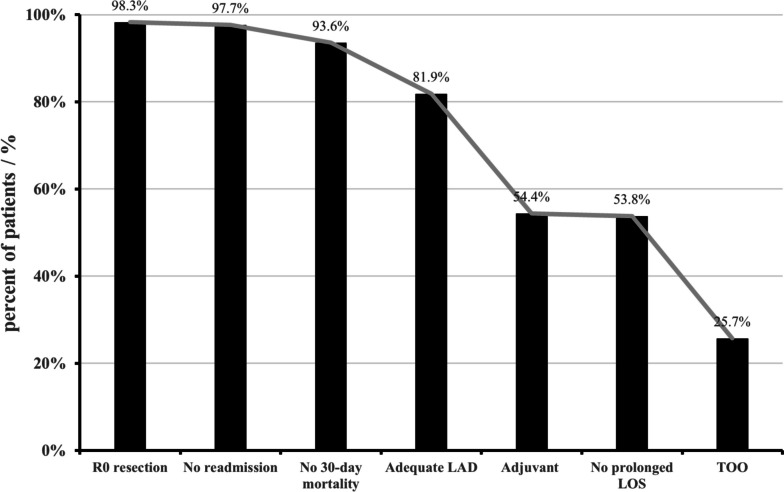
Textbook oncologic outcome (parameter and cumulative) after laparoscopic pancreaticoduodenectomy. LAD, lymphadenectomy; LOS, postoperative length of stay; TOO, textbook oncologic outcome

In total, 171 patients who met the inclusion criteria were enrolled. Among them, 44 (25.73%) patients achieved TOO. The results for the 6 individual outcome metrics are displayed in Fig. 1. The TOO outcome metric least frequently realized was “no prolonged LOS” (53.80%), followed by “receiving adjuvant chemotherapy within 12 weeks” realized in 54.39%, while the most frequently realized was R0 resection (98.25%).

The patients were divided into the TOO group and non-TOO group, and the baseline characteristics of patients in the two groups are shown in Table 1. Patients in the TOO group are younger than those in the non-TOO group (59.41 ± 9.54 vs. 63.42 ± 11.25 years,* P* = 0.036). Other preoperative data including gender, BMI, and the laboratory test did not show significant differences between the two groups (*P* > 0.05).

**Table 1:** Baseline characteristics of patients with or without achieving TOO after laparoscopic pancreaticoduodenectomy

Variables	TOO (*n* = 44, 25.73%)	Non-TOO (*n* = 127, 74.27%)	*P* value
Age (years)^a^	59.41 ± 9.54	63.42 ± 11.25	**0.036**
Sex (M) (*n* (%))	28 (63.63%)	76 (59.84%)	0.657
BMI (kg/m^2^)^a^	21.90 (20.24–22.74)	21.48 (19.91–23.11)	0.561
Hemoglobin (g/L)^a^	121.11 ± 16.42	123.38 ± 15.87	0.420
White blood cell (× 10^9^/L)^a^	6.27 ± 1.80	5.90 ± 2.37	0.333
Blood platelets (× 10^9^/L)^b^	207 (155–292)	212 (166–258)	0.791
Albumin (g/L)^a^	39.18 ± 4.90	38.16 ± 5.48	0.276
Creatinine (μmol/L)^b^	65.50 (55.50–77.50)	67.00 (59.00–78.00)	0.869
Total bilirubin (μmol/L)^b^	109.10 (13.78–223.05)	111.50 (15.70–228.40)	0.649
CA19-9 (U/mL)^b^	114.95 (46.41–284.28)	128.85 (52.93–322.75)	0.969
CEA (U/mL)^b^	3.09 (2.13–5.15)	3.59 (2.19–6.20)	0.833
Biliary drainage (*n* (%))	10 (22.73%)	29 (22.83%)	0.088

The bold value indicates statistical significance *P* < 0.05

*TOO* textbook oncologic outcomes, *non-TOO* textbook oncologic outcomes negative, *M* male, *BMI* body mass index, *CA19-9* carbohydrate atigen19-9, *CEA* carcinoembryonic antigen, *Y* yes

^a^Mean ± SD

^b^Median (IQR)

Table 2 shows the intraoperative and postoperative data of patients in the two groups. Patients in the TOO group had a significantly lower rate of DGE (6.82% vs. 24.41%, *P* = 0.011), and there was no significant difference in other intraoperative and postoperative data between the two groups.

**Table 2:** Intraoperative and postoperative data of patients with or without achieving TOO after laparoscopic pancreaticoduodenectomy

Variables	TOO (*n* = 44, 25.73%)	Non-TOO (*n* = 127, 74.27%)	*P* value
Tumor size (cm)^b^	3.00 (2.50–4.15)	3.00 (2.50–4.00)	0.337
OT (min)^a^	381.80 ± 82.96	387.36 ± 93.98	0.729
EBL (mL)^b^	150 (100–200)	200 (100–300)	0.989
Blood transfusion (*n* (%))	7 (15.91%)	17 (13.39%)	0.678
Diameter of MPD (mm)^b^	4 (3–6)	4 (3–5)	0.652
DGE (*n* (%))	3 (6.82%)	31 (24.41%)	**0.011**
CR-POPF (*n* (%))	0 (0%)	4 (3.15%)	0.234
PPH (*n* (%))	1 (2.27%)	4 (3.15%)	0.755
Infection of incisional wound (*n* (%))	0 (0%)	4 (3.15%)	0.203
Intra-abdominal infection (*n* (%))	0 (0%)	9 (7.09%)	0.067
PV/SMV resection (*n* (%))	12 (27.27%)	48 (37.80%)	0.208
Nerve invasion (*n* (%))	30 (68.18%)	94 (74.02%)	0.365
Vascular invasion (*n* (%))	14 (31.82%)	42 (33.07%)	0.564
Pathologic T stage (*n* (%))			
pT1	3 (6.82%)	21 (16.54%)	0.339
pT2	29 (65.91%)	81 (63.78%)	
pT3	12 (27.27%)	25 (19.69%)	
Pathologic N stage (*n* (%))			
pN0	31 (70.45%)	84 (66.14%)	0.415
pN1	10 (22.73%)	38 (29.92%)	
pN2	3 (6.82%)	5 (3.94%)	

The bold value indicates statistical significance *P* < 0.05

*TOO* textbook oncologic outcomes, *non-TOO* textbook oncologic outcomes negative, *OT* operation time, *EBL* estimated blood loss, *MPD* main pancreatic duct, *DGE* delayed gastric emptying, *CR-POPF* clinically relevant postoperative pancreatic fistula, *PPH* post-pancreatectomy hemorrhage, *PV/SMV* portal vein/superior mesenteric vein

^a^Mean ± SD

^b^Median (IQR)

### Survival analysis

**Fig. 2:**
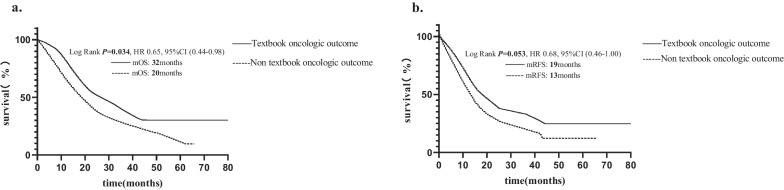
Kaplan‐Meier survival functions by receipt of the textbook oncologic outcome. **a** Survival curves for survival study population by receipt of the textbook oncologic outcome. **b** The disease-free survival curves study population by receipt of the textbook oncologic outcome. mOS, median overall survival; mRFS, median recurrence-free survival

The survival analysis of the two groups shows that TOO is associated with a survival advantage (32 vs. 20 m, *P* = 0.034) (Fig. 2a). Meanwhile, the two groups of patients in the RFS period also showed a certain difference, achieving TOO has longer RFS, although the difference did not reach statistical significance (19 vs. 13 m, *P* = 0.053) (Fig. 2b).

### Risk factors of TOO

Furthermore, Table 3 shows the univariate and multivariate logistic regression analysis of risk factors associated with TOO after LPD. Patients suffering from DGE [OR 4.045, 95% CI (1.151–14.214), *P* = 0.029] were independent risk factors for achieving TOO.

**Table 3:** Univariate and multivariate logistic regression for the criteria of textbook oncologic outcome achievement after laparoscopic pancreaticoduodenectomy

Patient variables	Univariate analysis		Multivariate analysis	
	**OR (95% CI)**	***P*** ** value**	**OR (95% CI)**	***P*** ** value**
Age (years)	1.034 (1.002–1.068)	**0.038**	1.024 (0.966–1.084)	0.436
Age > 65 (years)	2.109 (1.023–4.349)	**0.043**	1.157 (0.322–4.150)	0.823
Sex (male)	0.852 (0.419–1.731)	0.657		
BMI (kg/m^2^)	1.002 (0.984–1.021)	0.810		
BMI > 25 (kg/m^2^)	1.651 (0.527–5.176)	0.389		
Hemoglobin (g/L)	1.009 (0.987–1.031)	0.418		
Albumin (g/L)	0.965 (0.904–1.029)	0.275		
Creatinine (μmol/L)	0.999 (0.986–1.012)	0.868		
Total bilirubin (μmol/L)	1.001 (0.998–1.003)	0.647		
Biliary drainage (no)	1.904 (0.881–4.114)	0.101		
Tumor size (mm)	0.903 (0.670–1.217)	0.503		
OT (min)	1.001 (0.997–1.005)	0.727		
EBL (< 300 mL)	0.913 (0.355–2.348)	0.850		
Blood transfusion (yes)	0.649 (0.251–1.641)	0.361		
Diameter of MPD (< 3 mm)	0.581 (0.282–1.196)	0.140		
DGE (yes)	0.222 (0.064–0.767)	**0.017**	4.045 (1.151–14.214)	**0.029**

The bold value indicates statistical significance *P* < 0.05

*BMI* body mass index, *OT* operation time, *min* minutes, *EBL* estimated blood loss, *MPD* main pancreatic duct, *DGE* delayed gastric emptying

**Fig. 3:**
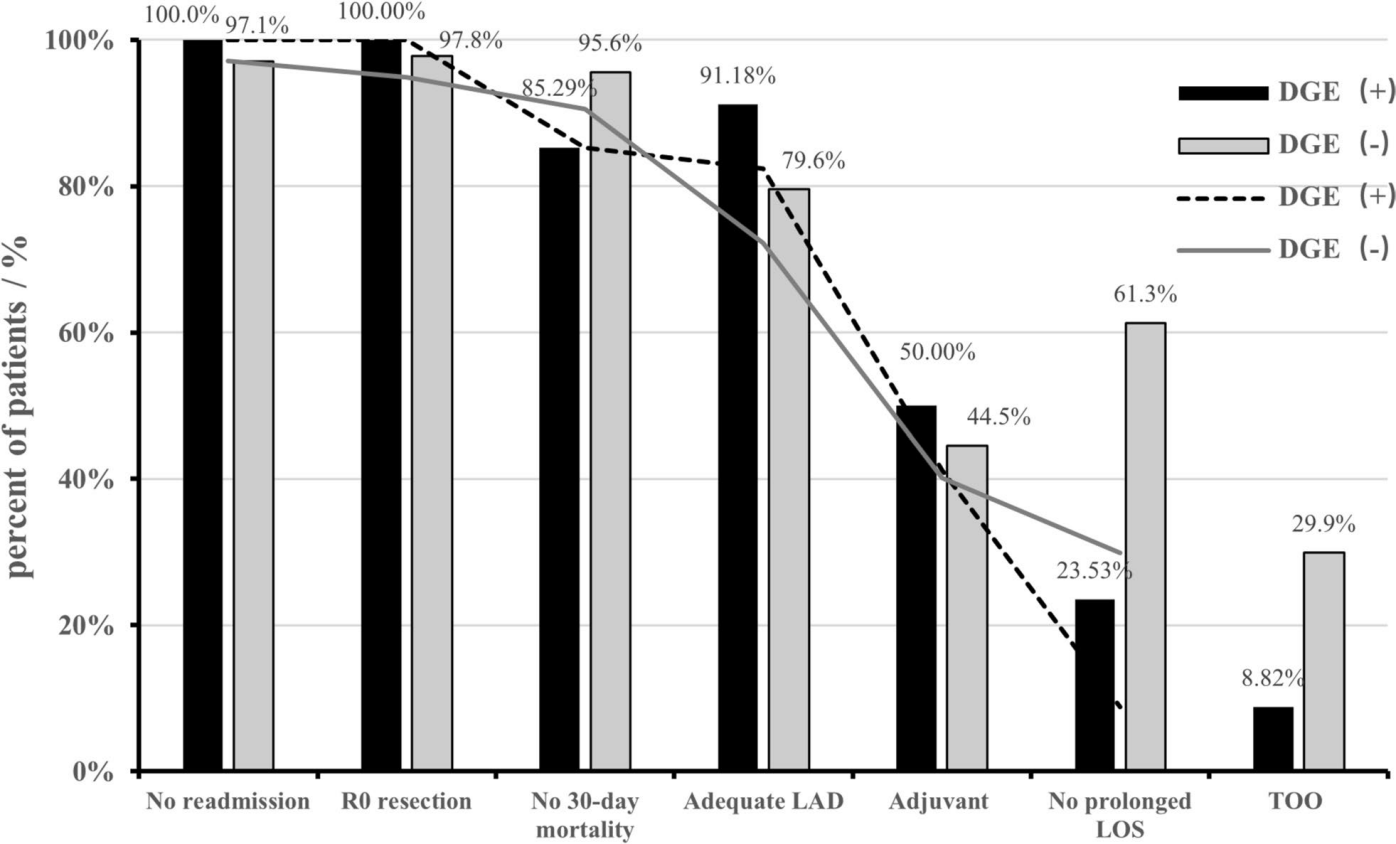
Textbook oncologic outcome (parameter and cumulative) after laparoscopic pancreaticoduodenectomy by group using DGE. LAD, lymphadenectomy; LOS, postoperative length of stay; TOO, textbook oncologic outcome; DGE, delayed gastric emptying

Only 3 patients (8.82%) with DGE achieved TOO, but 41 patients (29.93%) without DGE achieved TOO (Fig. 3). The rate of TOO has a significant difference between patients with and without DGE (*P* = 0.012), which is due to prolonged LOS (*P* < 0.001) and increased 30-day mortality (*P* = 0.028). The results can be found in Table 4.

**Table 4:** Analyze the effect of postoperative DGE on individual textbook oncologic outcome parameters

Characteristic	DGE ( +) (*n* = 34, 19.88%)	DGE ( −) (*n* = 137, 80.12%)	*P* value
No readmission	34 (100%)	133 (97.08%)	0.586
R0 resection	34 (100%)	134 (97.81%)	–
No 30-day mortality	29 (85.29%)	131 (95.62%)	**0.028**
Adequate LAD	31 (91.18%)	109 (79.56%)	0.140
Adjuvant	17 (50.00%)	61 (44.53%)	0.566
No prolonged LOS	8 (23.53%)	84 (61.31%)	** < 0.001**

*LAD* lymphadenectomy, *LOS* postoperative length of stay, *DGE* delayed gastric emptyings

## Discussion

TOO [26] is a comprehensive quality measure whose advantages include assessing the quality of surgery, predicting the long-term outcome of patients with digestive tract malignancies and measuring the burden of disease. To the best of our knowledge, this is the first study to evaluate TOO of patients with pancreatic head cancer who underwent LPD. In our study, we found that the achievement of TOO was significantly associated with the improvement of OS after LPD, which means that TOO can be used as a potential indicator for the comprehensive prognosis evaluation of surgical quality after LPD. The criteria of TOO mainly include early postoperative outcome indicators, which directly reflect the short-term results of patients. Several studies have examined the association between TOO and survival in cancer patients. Sweigert et al. found that achievement of TOO was associated with improved long-term survival in patients who underwent colectomy [10], and other researchers pointed out that a direct association exists between adjusted hospital TOO rates and survival after high-risk cancer procedures (eight types of malignant tumors were included) [26]. In addition, TOO has been found to be associated with survival in open pancreaticoduodenectomy [27–29]. In our study, achievement of TOO led to a significantly longer survival.

Meanwhile, the rate of TOO was 25.73% in our center. To our knowledge, although there are no relevant studies on TOO after LPD, previous studies reported that the rate of TOO after PD was less than a quarter. This is similar to the results of our study. For instance, the achievement rate of TOO in minimally invasive PD patients was 24.7% [5], and in the study by Sweigert and colleagues, TOO was only achieved in 16.8% of patients who underwent PD [27]. However, the rate of TOO was lower than in studies of other digestive tract malignancies. For example, 54.8% of patients who underwent colectomy achieved TOO [10]; the overall incidence of TOO was 69.0% among patients with liver cancer [11], and 37.2% of patients who underwent esophagectomy had achieved TOO [12]. The reasons for failure to achieve TOO also varied by cancer type. Aquina et al. conducted a comprehensive study of eight tumors, in which the study showed that patients with pancreatic cancer had the lowest TOO rate of 25%. The occurrence of this situation may be related to the higher degree of malignancy of pancreatic cancer. In this study, the main factors that hindered achieving TOO were receiving adjuvant therapy on time after surgery (54.39%) and prolonging the LOS (53.80%). This finding is similar to that of Sweigert et al. [27]. The main barriers hindered achieving TOO in other studies included R0 resection [26] and insufficient number of lymph nodes dissected [5]. In conclusion, the rate of TOO varies between different medical institutions.

Although the lower incidence of TOO in PDAC patients may be influenced by multiple factors, it may be likely to be related to the complexity of surgical procedures and the higher incidence of complications, which have been shown to prolong LOS and delay the delivery of adjuvant therapy [30, 31]. In our study, DGE after surgery was found to be an independent risk factor for TOO. In previous studies, DGE has been shown to prolong the LOS [31] and even lead to the risk of death [32]. This is consistent with our study. Otherwise, in other studies, risk factors affecting TOO after PD include age, race, economic ability, and the year of surgery [5, 27, 28, 33]. Since DGE was observed will affect the achievement of TOO, we divided patients into two groups according to with or without DGE and compared the trend of changes in each indicator of TOO. It is worth noting that the patients with DGE achieved a lower rate of TOO that was mainly attributed to prolonged LOS and increased mortality within 30 days. So, it is important to prevent the DGE after LPD to improve the rate of TOO.

Firstly, the main limitation of this study is its retrospective nature and a single-center study; information provided in digital medical records, follow-up, or differences in intraoperative techniques may increase the risk of bias. In our study, we have adopted many methods to minimize the bias caused by retrospective studies. For example, all data of this study was collected and checked retrospectively by two investigators from the prospective maintained medical record system, and any inconsistency needs to be discussed to get agreement. Meanwhile, the missing data was processed by sequential regression multiple imputation. Most importantly, we consecutively included all patients who met the inclusion criteria during the study period. Secondly, only a few patients underwent laparotomy, and we only enrolled patients with LPD so that it will limit the generalizability of the findings. Thirdly, due to the combined effect of the inclusion and exclusion criteria, no patients after neoadjuvant chemotherapy were included in this study; therefore, the rate of TOO may be lower than reported.

## Conclusion

In conclusion, TOO is a good indicator for long-term oncologic outcomes in patients with pancreatic head cancer after undergoing LPD. DGE is the risk factor for achieving TOO, and it is important to prevent the DGE after LPD.

## Data Availability

No datasets were generated or analysed during the current study.
